# Prepatellar bursa mucosa: An unreported postoperative complication of patellar fracture

**DOI:** 10.1097/MD.0000000000040445

**Published:** 2024-11-08

**Authors:** Fukun Lin, Jihang Yao, Yiming Liu, Baochang Qi

**Affiliations:** a Department of Orthopedic Traumatology, The First Hospital of Jilin University, Changchun, China.

**Keywords:** complication, operation, patellar fracture, prepatellar bursa mucosa

## Abstract

**Background:**

Patellar fracture is a common injury caused by overstrain of the extensor mechanism or a direct trauma to the knee. The most common complications after patellar fracture are nonunion, infection, post-traumatic arthritis, joint fibrosis, symptomatic hardware, and extensor mechanism failure. These are attributed to the post-fracture flexion and extension movements, the primary damage to the articular cartilage, and the thin soft tissue coverage over the knee. To our knowledge, prepatellar bursa mucosa is an unreported postoperative complication of patellar fracture.

**Methods:**

We describe a case of a 58-year-old woman with a left patellar fracture from a fall. Complete healing was achieved after in 2 months postoperatively using the improved Kirschner wire tension band and a cable grip system; however, at 7 months postoperatively, a prepatellar skin mass appeared. The patient reported prepatellar discomfort with no pain and influence of movement of the knee and daily life. One year postoperatively, we surgically removed the implants and mass. The pathological result showed bursa mucosa.

**Results:**

The literature review shows that pain, secondary knee dysfunction, fracture nonunion, and malunion are postoperative complications of patellar fractures. However, prepatellar bursa mucosa have not been previously reported as a postoperative complication.

**Conclusion:**

This case highlights the need for appropriate placement of the intraoperative implant in adults with patellar fractures.

## 1. Introduction

Patellar fracture is relatively rare compared with other lower limb fractures and accounts for only 1% to 1.65% of adult fractures, 1/3 of which are comminuted fractures. However, with the development of transportation and construction industry in recent years, the number of patients with patellar fracture has been increasing.^[1,2]^ The patella increases power and mechanical advantage of the extensor mechanism by 30% to 50% by displacing it anteriorly away from the center of rotation. During knee flexion, the patella experiences tension from the quadriceps and patellar tendon and compressive loads across posterior patella. Due to the critical function of the lower limb extensor mechanism, the most common complications after patellar fracture may be nonunion, infection, post-traumatic arthritis, joint fibrosis, symptomatic hardware, and extensor mechanism failure. At the early stage, which is perhaps within 4 weeks postoperatively, we suggest patients to do flexion and extension exercises, which will bring good function, but they may also increase the risk of nonunion, infection, and hardware failure (extensor mechanism failure) because of the movements. The primary damage of the patellar articular cartilage can lead to complications of post-traumatic arthritis and joint fibrosis. The thin soft tissue coverage of the patella will lead to symptomatic hardware. To our knowledge, prepatellar bursa mucosa (the bursa mucosa located anterior to the patella) is an unreported complication of patellar fracture.^[3]^ We herein report on surgical treatment of patellar fracture with an unreported complication of prepatellar bursa mucosa after fixation.

## 2. Case report

**Figure 1.**
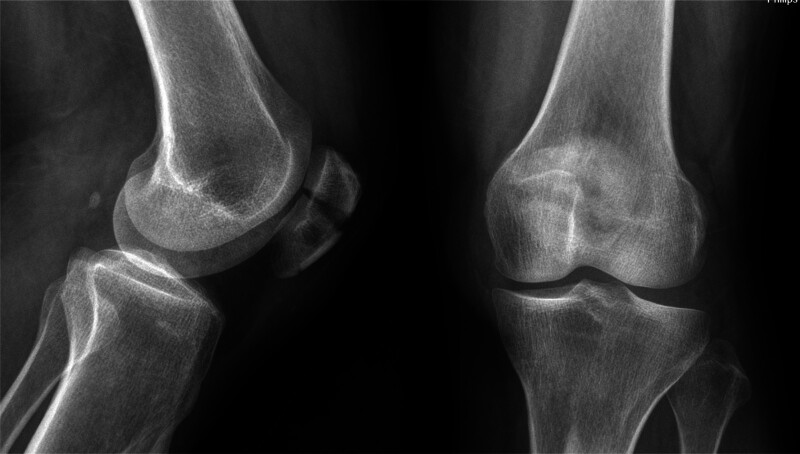
Imaging findings of the patient at the time of injury.

**Figure 2.**
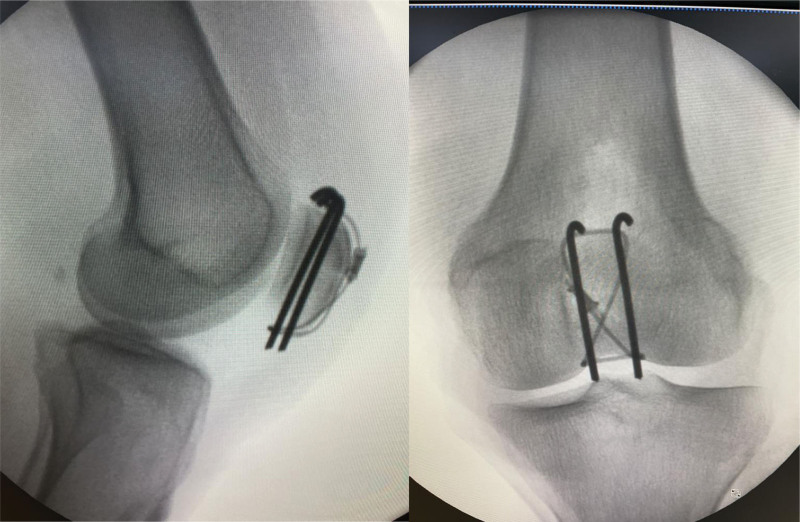
Intraoperative fluoroscopy showed that the fracture alignment was good.

**Figure 3.**
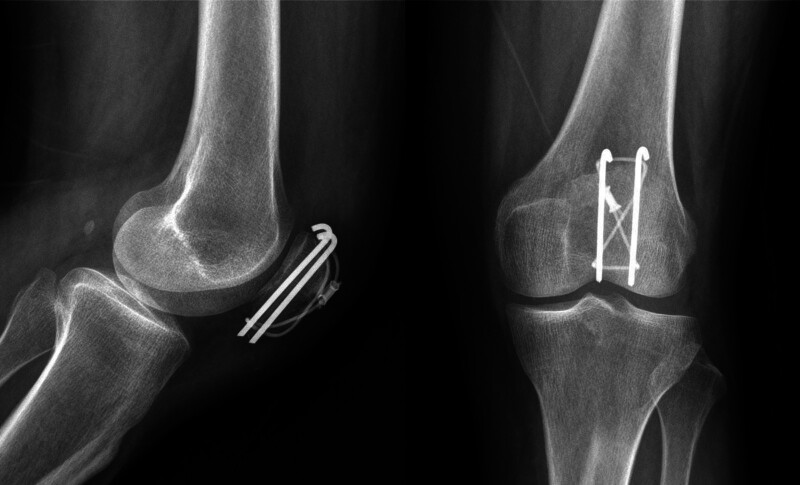
The 1 month postoperative X-ray film showed satisfactory reduction and fixation of the patellar fracture.

A 58-year-old woman was brought to the emergency room after a fall. Her radiographs showed a fracture of the left patella (Fig. 1). She was 155 cm tall and weighed 50 kg. Fractures were classified as AO/OTA 34C1.1. She was operated on using a figure-of-eight tension band^[4]^ by Kirschner wire and a cable grip system (Cable-Ready ZIMMER BIOMET, Warsaw), and the fracture site alignment was identified as good under C-arm fluoroscopy (Fig. 2). Her 1-month postoperative radiographs showed satisfactory reduction and fixation of the left patella (Fig. 3).

**Figure 4.**
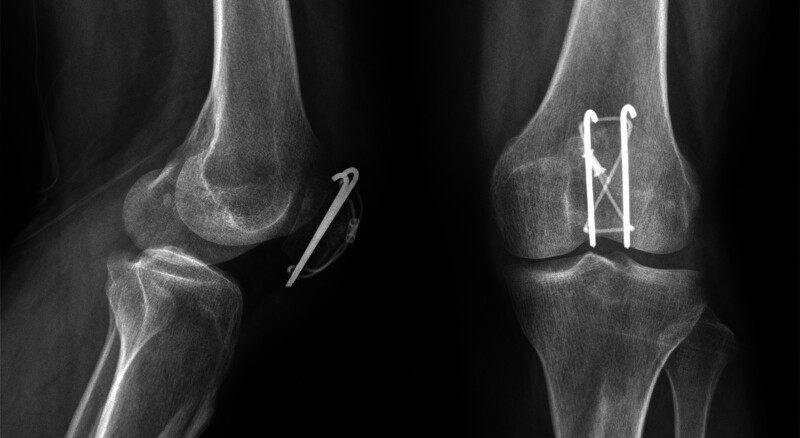
Imaging reports showed that the alignment of the fracture site was good; the internal fixation did not shift at 2nd month postoperatively.

**Figure 5.**
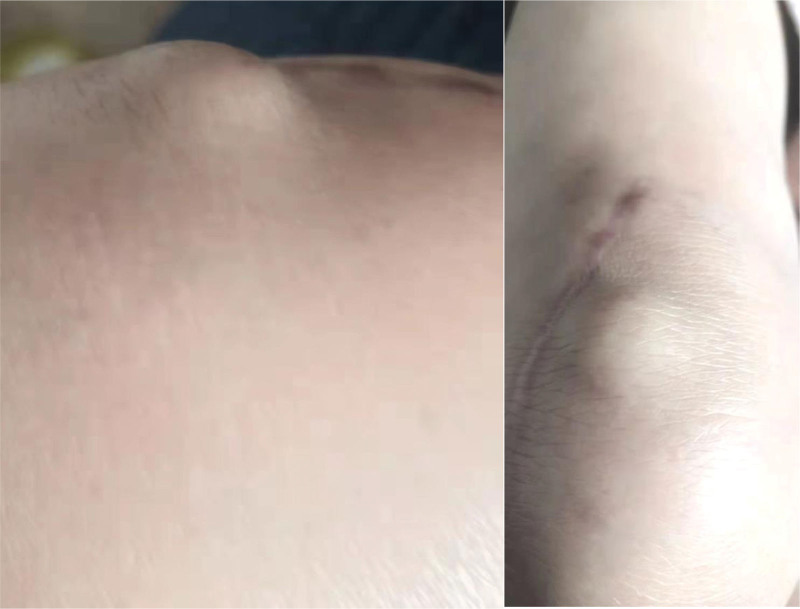
Slight eminence of the prepatellar skin during flexion and extension of the knee joint at 7th month postoperatively.

**Figure 6.**
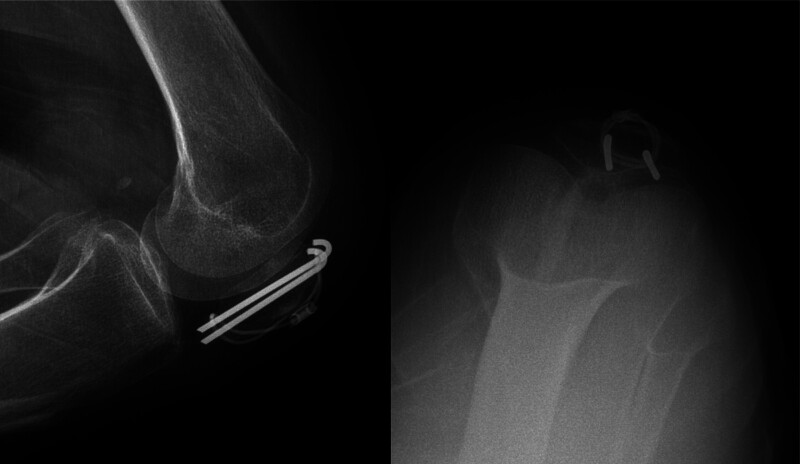
X-ray results showed that the fracture had healed very well without hardware failure at 7th month postoperatively.

**Figure 7.**
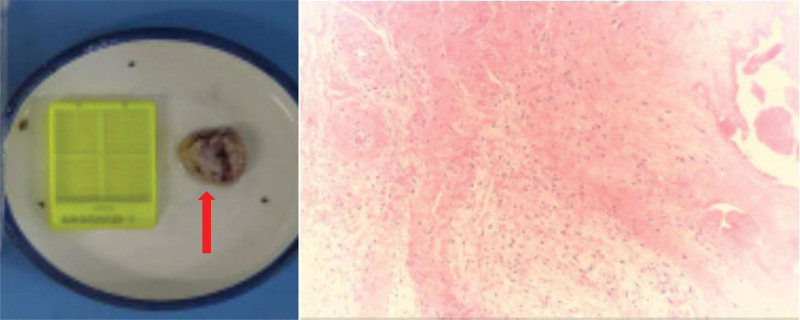
The pathological result showed bursa mucosa.

The patient performed normal knee functional exercises under the supervision of the doctor after 3 days after the surgery. The second month after the surgery, the patient came to the hospital for her scheduled follow-up visit. Imaging reports revealed good alignment of the fracture site; the internal fixation did not shift (Fig. 4), and she could perform functional exercises of the affected limb well. At 7 months postoperatively, the patient reported prepatellar discomfort and slight eminence of the prepatellar skin during flexion and extension of the knee joint (Fig. 5). X-ray findings showed that the fracture had healed very well without hardware failure (Fig. 6). Physical examination of the operated knee showed that the skin over the affected side of the anterior patella was raised and moved freely, and she reported feeling no pain during movement of the knee. Her implants and the mass were surgically removed 1 year postoperatively. The pathological result showed bursa mucosa (Fig. 7). The patient reported no discomfort and good function of the left knee after the operation.

We suspect that the prepatellar bursa mucosa formation is due to soft tissue wear caused by the fixation of the cable grip system clip (Cable-Ready ZIMMER BIOMET) and that the body formed the bursa mucosa for protective mechanisms. After this patient, we paid much more attention to the clip position of the cable grip system during each patellar fracture operation so that the clip was not centered. No similar symptoms were found in 25 consecutive patients.

## 3. Discussion

Despite the patellar fracture accounting for 1% of all fractures, its functional outcome has been neglected in the literature. At present, the relatively common types of patellar fracture are AO/OTA and Rock-wood. The AO/OTA classification is as follows: Type A: patellar extra-articular fracture; Type B: partial intra-articular fracture of the patella but normal knee extensor; and Type C: complete intra-articular fracture of the patella and fracture of the knee extensor.^[4,5]^ The unreported complications in this case highlight the significance of implant placement of the cable grip system for intraoperative fracture fixation, and the surgeon’s attention in this regard during the operation may avoid some unnecessary complications.

Although prepatellar subcutaneous bursa mucosa does not greatly impact the patient’s quality of life, it impacts knee joint activity in the early stage of rehabilitation training. The discomfort may affect postoperative functioning of the knee. Moreover, prepatellar subcutaneous bursa mucosa leads to unfavorable cosmetic appearance of the knee, which is unacceptable for young patients, particularly young women. Therefore, proper intraoperative placement of the clip position of the cable grip system is very important.

Patellar fractures are not particularly rare, and therefore, the assessment of treatment and outcomes is challenging, particularly as there is no standardized rehabilitation regimen.

## 4. Conclusion

We report this case because it illustrates a complication of patellar fracture fixation that has not been previously described or routinely recognized. In addition, we herein emphasize the fundamental importance of proper intraoperative placement of internal fixators and postoperative follow-up visits.

## Author contributions

**Conceptualization:** Baochang Qi.

**Investigation:** Yiming Liu.

**Writing – original draft:** Fukun Lin.

**Writing – review & editing:** Jihang Yao.
